# Machine Learning–Based Hospital Discharge Prediction for Patients With Cardiovascular Diseases: Development and Usability Study

**DOI:** 10.2196/32662

**Published:** 2021-11-17

**Authors:** Imjin Ahn, Hansle Gwon, Heejun Kang, Yunha Kim, Hyeram Seo, Heejung Choi, Ha Na Cho, Minkyoung Kim, Tae Joon Jun, Young-Hak Kim

**Affiliations:** 1 Department of Medical Science Asan Medical Institute of Convergence Science and Technology Asan Medical Center, University of Ulsan College of Medicine Seoul Republic of Korea; 2 Division of Cardiology Department of Internal Medicine Asan Medical Center, University of Ulsan College of Medicine Seoul Republic of Korea; 3 Big Data Research Center Asan Institute for Life Sciences Asan Medical Center Seoul Republic of Korea

**Keywords:** electronic health records, cardiovascular diseases, discharge prediction, bed management, explainable artificial intelligence

## Abstract

**Background:**

Effective resource management in hospitals can improve the quality of medical services by reducing labor-intensive burdens on staff, decreasing inpatient waiting time, and securing the optimal treatment time. The use of hospital processes requires effective bed management; a stay in the hospital that is longer than the optimal treatment time hinders bed management. Therefore, predicting a patient’s hospitalization period may support the making of judicious decisions regarding bed management.

**Objective:**

First, this study aims to develop a machine learning (ML)–based predictive model for predicting the discharge probability of inpatients with cardiovascular diseases (CVDs). Second, we aim to assess the outcome of the predictive model and explain the primary risk factors of inpatients for patient-specific care. Finally, we aim to evaluate whether our ML-based predictive model helps manage bed scheduling efficiently and detects long-term inpatients in advance to improve the use of hospital processes and enhance the quality of medical services.

**Methods:**

We set up the cohort criteria and extracted the data from CardioNet, a manually curated database that specializes in CVDs. We processed the data to create a suitable data set by reindexing the date-index, integrating the present features with past features from the previous 3 years, and imputing missing values. Subsequently, we trained the ML-based predictive models and evaluated them to find an elaborate model. Finally, we predicted the discharge probability within 3 days and explained the outcomes of the model by identifying, quantifying, and visualizing its features.

**Results:**

We experimented with 5 ML-based models using 5 cross-validations. Extreme gradient boosting, which was selected as the final model, accomplished an average area under the receiver operating characteristic curve score that was 0.865 higher than that of the other models (ie, logistic regression, random forest, support vector machine, and multilayer perceptron). Furthermore, we performed feature reduction, represented the feature importance, and assessed prediction outcomes. One of the outcomes, the individual explainer, provides a discharge score during hospitalization and a daily feature influence score to the medical team and patients. Finally, we visualized simulated bed management to use the outcomes.

**Conclusions:**

In this study, we propose an individual explainer based on an ML-based predictive model, which provides the discharge probability and relative contributions of individual features. Our model can assist medical teams and patients in identifying individual and common risk factors in CVDs and can support hospital administrators in improving the management of hospital beds and other resources.

## Introduction

### Background

The use of human and physical resources, which are both costly and scarce, is essential for the efficient operation of hospital processes. Hospitals are required to manage different kinds of resources, such as managing the schedules of the medical team and staff, bed management , and clinical pathways to improve overall management efficiency [1]. Effective resource management in hospitals can improve the quality of medical services by reducing the labor-intensive burden on staff, decreasing inpatient waiting time, and securing optimal treatment time [2].

Bed management is a form of hospital resource management. Currently, in most hospitals, clinicians manually check a patient’s condition to decide whether to continue their hospitalization or discharge them [3]. On the basis of this decision, the medical team and staff identify the bed capacity available in the near future and schedule the patient’s reservation. In addition, the number of patients hospitalized for a variety of chronic and acute illnesses, such as cardiovascular diseases (CVDs) [4], has been steadily increasing, and their insufficient treatment can lead to readmissions or complications. However, a stay in the hospital longer than the optimal treatment time hinders effective bed management. Thus, it is important to accurately predict the patient’s hospitalization period and make judicious decisions about their discharge.

Many studies have focused on the efficiency of hospital resources, and most of them presented algorithms or models for improving bed management. Bachouch et al [5] investigated hospital bed planning and proposed the integer linear program to solve the optimization problem. They illustrated the simulated bed occupancy schedule. Troy et al [6] studied the simulation of beds for surgery patients using the Monte Carlo simulation to determine the intensive care unit (ICU) capacity. Particularly, the predicted length of stay (LOS) is one necessary piece of information for bed management, and there are many studies predicting the LOS based on electronic health records (EHRs) [7-9].

Moreover, authors have used machine learning (ML)–based models to predict the LOS [7-9], prolonged hospitalization, and unplanned readmission [10] and to find biomarkers for critical diseases [11]. Recently, there have been many studies on interpretable or explainable artificial intelligence (XAI) [12]. One XAI study [13] developed a model to predict acute illness and provide results and interpretation. Compared with EHRs, studies employing computer vision algorithms such as convolutional neural networks are more actively pursued because these models can directly visualize significant parts of an image [14,15]. Thus, we developed an ML-based predictive model to provide the daily discharge probability and *individual explainer* visualizing significant features of each patient to support bed management.

### Objectives

The main contributions of this study can be summarized in the following steps: first, we developed an ML-based predictive model to predict the discharge probability daily within 3 days for each patient with CVD and to acquire the individual LOS. Patients with chronic and acute diseases, including CVDs, have high hospitalization and readmission rates and greater complications [16]. There are alternatives to transfer those who need urgent care or hospitalization to another hospital to address delays. However, it could be causing other serious problems, hospitals should continuously identify methods to reduce waiting time, and efficient bed management can be considered as one of them.

In addition, because of the diversity of diseases, it may be more advantageous to find common risk factors and implement bed management for specific departments or diseases (ie, clustered specific wards), and then expand it further to the hospital level. Therefore, we developed an ML-based model to determine the bed capacity that would be available in the near future and find risk factors by predicting the discharge of patients hospitalized with CVDs [17]. By providing persuasive discharge information such as expected individual discharge date and risk factors related to CVDs, it is possible, in practice, to assist in precise bed management, which is otherwise done manually by the medical team.

Second, we assessed the outcome of the prediction and provided the individual explainer to describe the primary risk factors of inpatients for patient-specific care. Even if patients have the same diseases and common variables represent the diseases, each patient has different characteristics, history, circumstances, and treatments. Therefore, it is also necessary to identify and monitor the unique, individual variables for each patient. In this study, our ML-based predictive model’s outcomes include not only information on daily patient discharge but also the contributions of features such as feature importance. Furthermore, we visualized the day-by-day discharge probability of each patient and the features that influenced individual patients during the hospitalization. This explainer can guide the medical team and patients to produce reasonable evidence on the ML-based model’s outcomes and helps them understand the conditions in detail and prepare in advance for treatment. Such individual analysis can focus on each patient, and the meaningful features identified can be used in other studies as a basis for preidentifying variables affecting hospitalization.

Third, this study could help manage bed scheduling efficiently and detect long-term inpatients in advance. Bed management refers to the process of identifying patients who are most likely to be discharged, confirming the number of available beds, and allocating beds to patients waiting for admission after reservation. As this process is complicated and usually carried out manually, we aimed to support it by providing the estimated LOS and probability of discharge returned by the model and by identifying the capacity of beds that would be available in the near future. In addition, it is possible to detect not only patients with a high probability of discharge but also patients with a consistently low probability of discharge. In other words, it helps discover and analyze the causes of long-term hospitalization of high-risk patients and provides this information to their management team.

**Figure 1:**
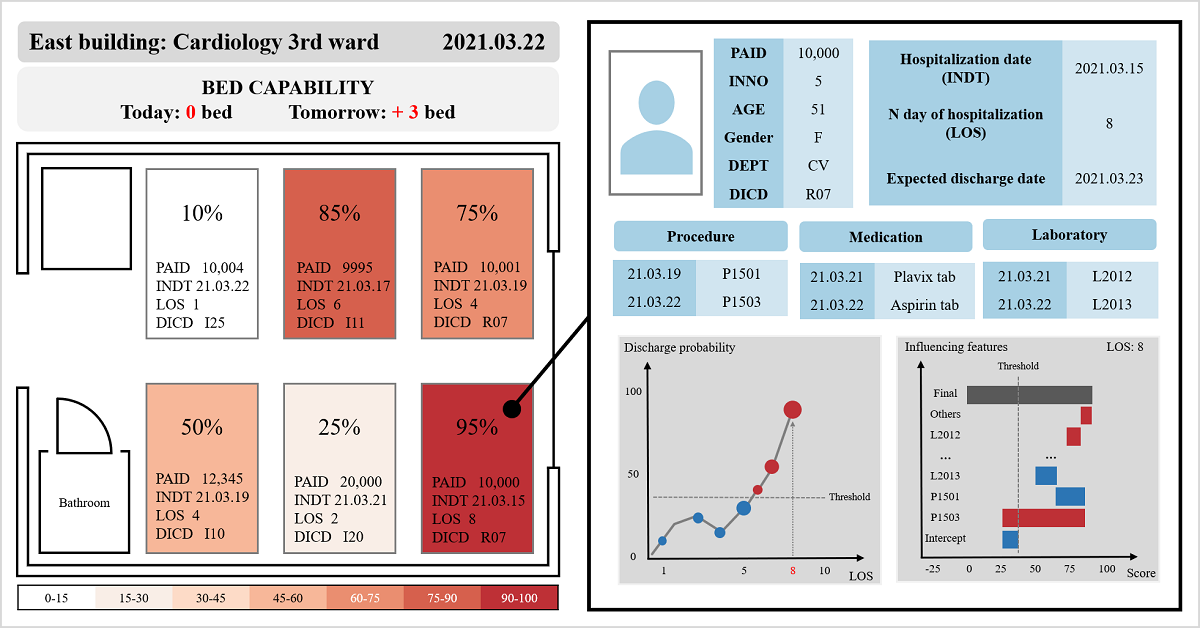
Visualized simulation of discharge prediction for machine learning–based bed management. DEPT: department; DICD: diagnostic code; INDT: the date of visitation or admission; INNO: the patient’s encounter number; LOS: length of stay; PAID: the patient’s identification.

To summarize, we developed an ML-based model to predict whether hospitalized patients with CVDs would be discharged within 3 days. On the basis of this model, we proposed an individual explainer; the simulations of bed management are depicted in Figure 1, including the probability of discharge and influenced features such as demographics, prescribed medications, and treatments. Our model can improve the efficient use of hospital resources and enhance the quality of medical services.

## Methods

### Overview

**Figure 2:**
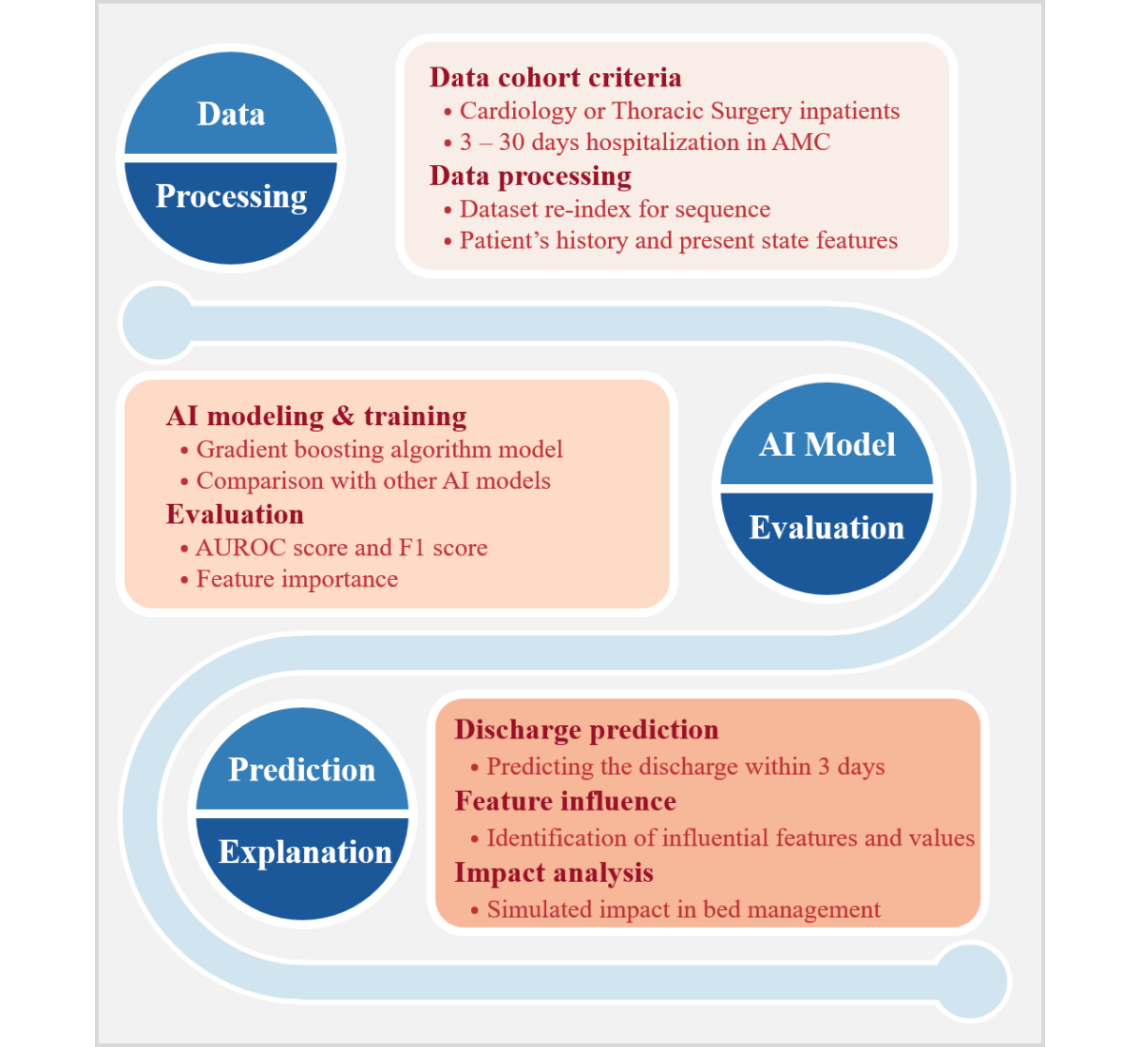
Overall flow of the prediction method for discharge within 3 days. AI: artificial intelligence; AMC: Asan Medical Center; AUROC: area under the receiver operating characteristic.

Figure 2 describes the overall flow of the prediction method employed in this study. We set up the cohort criteria and processed the data to create suitable data sets. Subsequently, we trained the ML-based predictive models and evaluated them to find an elaborate model. Finally, we predicted the discharge probability within 3 days and explained the model’s outcomes by identifying, quantifying, and visualizing its features.

### Data Acquisition

Data were extracted from CardioNet [18] (Textbox 1), a manually curated EHR database specialized in CVDs. CardioNet consists of data from 572,811 patients who had visited Asan Medical Center (AMC) with CVDs between January 1, 2000, and December 31, 2016. The AMC institutional review board approved the collection of CardioNet data and waived informed consent. CardioNet contains 27 tables on topics such as visitation, demographics, diagnosis, medication, and laboratory examination. Most tables have common variables including patient identification (PAID), patient encounter number (INNO), the date of visitation or admission (INDT), and the date of discharge (OUDT). The KEY column, which concatenates the PAID and INNO columns, can connect the visitation table to other tables. Using the KEY column, we extracted the variables in each table to be analyzed.

From the 572,811 patients in CardioNet, we obtained 84,251 records of 63,261 anonymous patients hospitalized in the departments of cardiology or thoracic surgery. Furthermore, to develop a practical and usable model, we focused on predicting discharge within 3 days and detecting long-term patients. Long-term patients, defined as those hospitalized for more than 30 days, are separately managed by the AMC. Therefore, we set the LOS between 3 and 30 days.

**Data extracted from CardioNet.**

- Visit table: patient identification, patient encounter number, KEY, date of visitation or admission, date of discharge, type of visit, medical department, and duration of stay in the intensive care unit (ICU).
  acute care unit, coronary care unit, cardiac surgery ICU, medical ICU, neonatal ICU, neurological ICU, neurosurgical ICU, pediatric ICU, and surgical ICU.
- Diagnosis table: *International Classification of Diseases, Tenth Revision* code of diagnosis.
- Laboratory test result table: date and code of pathology examination, and the result of the examination.
- Physical information table: patient’s age, height, weight, systolic and diastolic blood pressures, respiratory rate, pulse rate, BMI, body surface area, and date of measurements.
- Medication table: date and code of prescription.
- Procedure table: date and code of order.
- Operation table: date and code of surgery or treatment.
- Picture archiving and communication system table: date and code of order.
- Transfusion order table: date and code of order.

### Data Preprocessing

#### Data Set Creation

In the visit table, which is the primary table of CardioNet, there are 4 main columns (PAID, INNO, INDT, OUDT) and visit-related variables. Each row represents a single hospitalization case for each inpatient. We reset the index to create a new data set with the duration between admission and discharge as date-index (eg, a row with an INDT of 2021.2.1 and an OUDT of 2021.2.10 has an LOS 10 of days; therefore, it was converted to 10 rows with 10 date-indexes). Finally, after preprocessing all values corresponding to PAID, INNO, and date-indexes of other tables, we merged and concatenated the tables to generate a new data set for model training.

**Figure 3:**
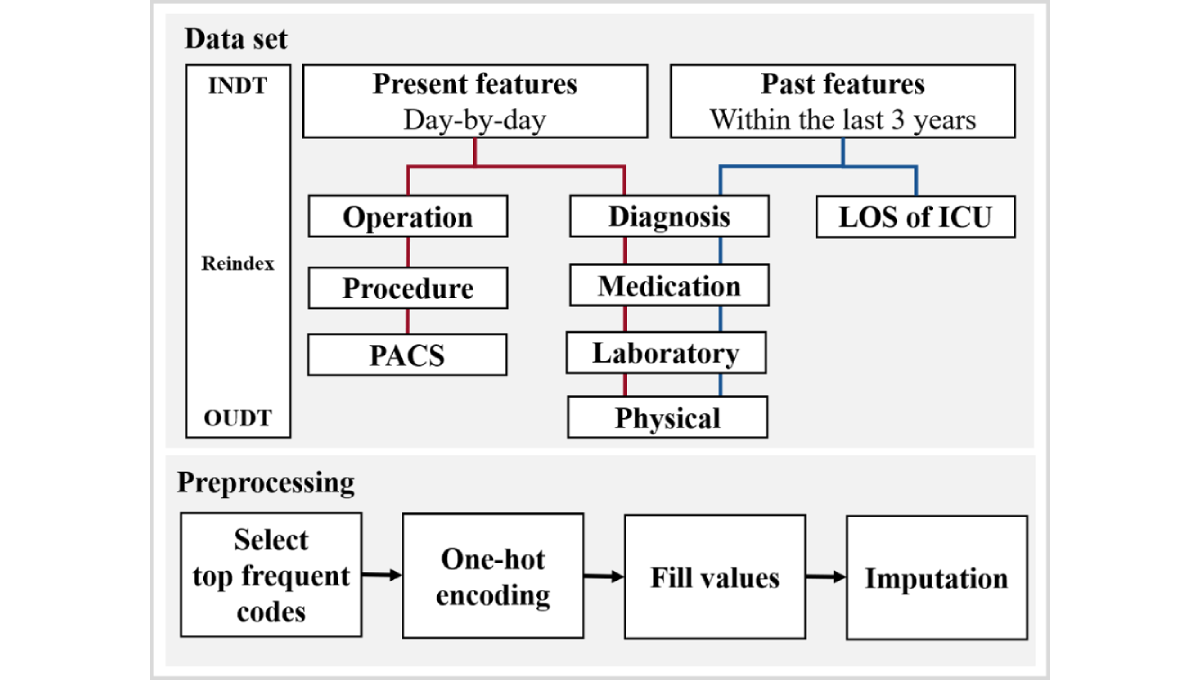
Data set creation process for machine learning–based model training. ICU: intensive care unit; INDT: date of visitation or admission; LOS: length of stay; OUDT: date of discharge; PACS: picture archiving and communication system.

Figure 3 shows the data set creation process. Each table of diagnosis, medication, laboratory test results, and physical information was used for both past and present features. The operation, procedure, and picture archiving and communication system (PACS) were used for the present features, and LOS in the ICU was used for the past features. The preprocessing of values for each table is discussed in the next section. The specific methods of feature handling are as follows:

#### Data-Related Features

After creating the new data set, we removed the OUDT containing future information. To distinguish and recognize the time information in date by type, we created a total of 10 date-related features. INDT and date-index were sliced into integer features such as year, month, day, and weekday. Furthermore, we created a feature that denotes whether the date-index is a holiday or not and another feature that indicates the LOS at the date-index by subtracting INDT from the date-index.

#### Day-by-day Present Features Related to Hospitalization

As the visit table and other tables contain only one piece of information per row, it is difficult for the ML model to learn the data all at once. Therefore, we performed one-hot encoding (OHE) of clinically important orders and codes and created them as features in the new data set. Consequently, we could access aggregated records by date for each patient.

First, in the diagnosis and operation tables, we sliced all the values of the International Classification of Diseases-10th edition codes and the operation codes at the third digit to convert them into three-digit codes because the strings from the fourth digit onward represent the subhierarchy of the three-digit codes. We arranged all the frequency values in descending order and selected the first 99 codes. We transformed the remaining codes (ie, unselected codes) into the *others* feature and performed the OHE on all 100 codes. The features in the form of *Z_code*, such as *Z_DICD* and *Z_OPCD*, refer to *others* in each original table. As a result, we obtained a total of 100 codes for each table (ie, diagnosis and operation table) and filled the date-index values with *1* if there were valid prescribed or ordered data and *0* otherwise. Similarly, the values of the PACS table were converted to 100 features.

Second, similar to the diagnosis table, in the medication and procedure tables, we obtained the 99 most frequent codes and *others*, performed the OHE, and filled the corresponding data. In the case of the transfusion table, we used all 27 codes available. We filled the values with the number of prescriptions per day or at once, considering the severity of each patient’s ailment.

Third, in the laboratory test result table, the 60 most frequent examination codes, examined in more than 50% of all patients, were selected. The physical information table had only 10 codes, which were all used. We performed the OHE of values and filled them with results corresponding to each examination. If a patient had been tested several times a day, the data set was populated with the average of the results.

#### Past Features

We considered that the patient’s anamnesis (ie, medical history) should also be included in the data set, along with the day-to-day features (described in the previous paragraph) for the ML model to learn the data deeply. When the date-index in each hospitalization started from INDT, we created some past features from the principal information of hospital visit records 3 years before INDT.

For past features, OHE was performed, and values were filled in, similar to the present features. The hospitalization periods of all ICUs in the visit table were summed up. For 100 diagnostic codes, we summed up each value if there was a record of diagnosis. For 100 medication codes, the number of prescriptions per day or at once were summed up if the record existed. Finally, recent laboratory test results and physical information within 3 years were used for a total of 70 codes. In conclusion, the data set was filled with either summed up or recent values equivalent to each feature.

#### Imputation

Except for the laboratory, physical, and date-related features, we replaced all the null values with zero. The value type of most of the other features was null or integer because most were calculated by frequency. In contrast, to deal with missing values in the continuous data type of the present laboratory and physical features, we first separated the data set based on the KEY. The KEY refers to a single hospitalization case of one patient; thus, separating the data set by KEY does not mix individual hospitalizations. Therefore, we filled in null values in chronological order (ie, from past to present). Subsequently, we filled in the rest of the null values in reverse chronological order (ie, from present to past) to handle those cases where results were not measured at the beginning of the admission. Using this method, it was possible to impute the null value for each hospitalization of an individual patient. Finally, to fill the values where all the features were not ordered or measured, we filled the rest of the null values with the most frequent value for each feature.

#### Target Criteria

**Figure 4:**
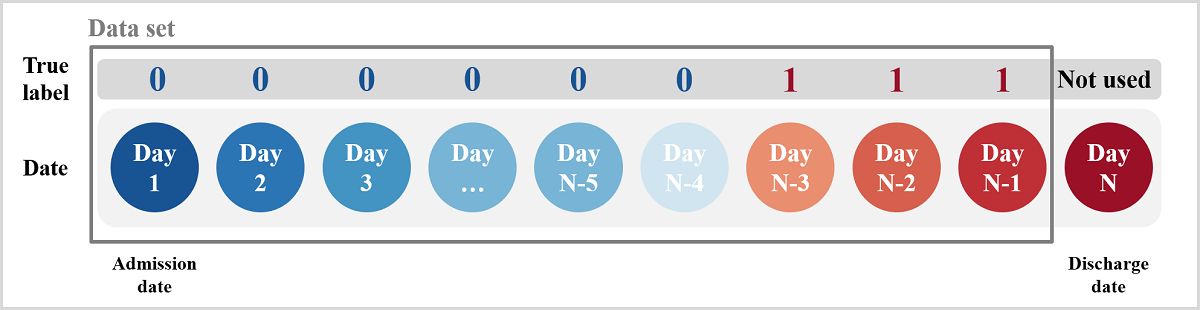
Target criteria to provide the true label (ie, correct answer) to machine learning–based models.

The supervised learning algorithm for classification requires the label *true* or *false* to indicate the correct answer. The target criteria for *true* labeling in this study are depicted in Figure 4.

As shown in Figure 4, *day 1* is INDT, *day N* is OUDT, and the circles represent each day of the hospitalization period. We excluded *day N* (ie, discharge date) from the data set because of information such as *discharge procedure*, which could provide the ML model with a hint. In addition, even if the accuracy of discharge prediction is higher from the discharge date to 2 days earlier, it is useful to make the prediction 3 days in advance when actually using the model. Therefore, we labeled *1* from one day before OUDT to 3 days before OUDT and labeled *0* from the INDT to 4 days before OUDT.

As a result, we transformed the diverse variables of original tables into 10 date-related features, 597 present features, and 279 past features, creating a data set of 669,667 rows with 886 features from 84,251 records of 63,261 inpatients with CVDs.

### ML-Based Predictive Models

#### ML-Based Models

We experimented with 5 models to identify the most suitable one. We set the logistic regression [19] model as the baseline to estimate performance, and support vector machine [20,21], random forest (RF) [22], multilayer perceptron (MLP) [23], and extreme gradient boosting (XGB) [24] were selected as comparison models. We also performed hyperparameter tuning for each model through random search.

We selected XGB, which is a gradient boosting algorithm (GBM) model, as the final model. GBM is an ensemble method that combines several weak classifiers (trees). The main idea of GBM is to focus and place the weights on incorrectly predicted results. While XGB is training, one tree trains the data set and assigns weights to incorrectly predicted records with errors, and the next tree of the same model learns the weighted data set and repeats the process of assigning weights. Moreover, GBM can quantify the contribution of features to the prediction results, such as feature importance. Particularly, XGB has the advantage of regularization and performance. It can perform parallel processing, regulate to avoid overfitting, is widely used for learning structured data, and has superior prediction performance.

#### Evaluation

We set the positive (1) label for discharge and the negative (0) label for hospitalization. To evaluate and compare the performance of candidate models, we used metrics including accuracy, sensitivity (recall for positive), specificity, precision, positive predictive value, negative predictive value, false-positive rate, and true-positive rate. When we monitored model training and validation, we used the F1-score to reflect imbalanced targets, the receiver operating characteristic (ROC) curve to find the optimal threshold, and the area under the ROC (AUROC) score to compare models.

**Figure 5:**
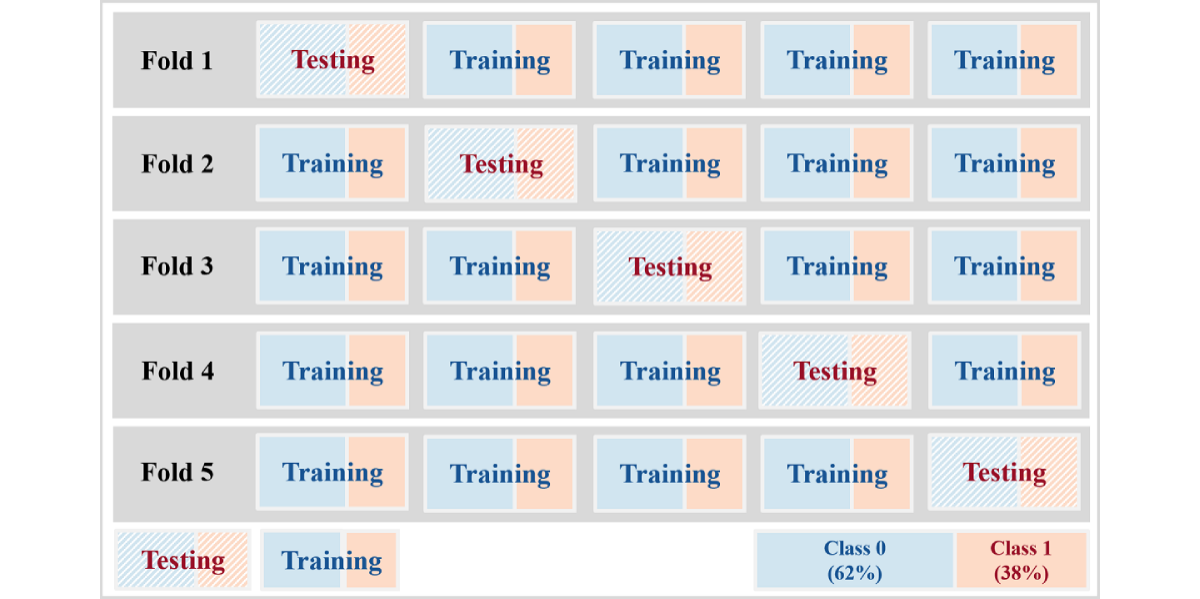
Stratified 5-fold cross-validation to avoid overfitting.

To prevent overfitting the ML-based models and reduce biased results, we performed stratified, 5-fold cross-validation [25] illustrated in Figure 5. First, we randomly shuffled 63,261 PAIDs and divided them into 5 groups with approximately 12,000 people because we tried not to divide the records of a single patient into training (ie, plain box in Figure 5) and testing sets (ie, diagonal hatching box in Figure 5). Second, the first PAID group becomes the testing set, and the remaining groups become the training set in fold 1. We created fold 1 to fold 5 in a similar way to ensure equal division of the imbalanced targets (ie, the data set has true labels comprising 62.4% label *0* and 37.6% label *1*) across all folds. Besides, we split 25% of the training set as the validation set to tune the hyperparameters. Consequently, in each fold, we divided the data set into approximately 133,000 rows for the testing set and 535,000 rows for the training set (including the validation set). The ML-based models trained and tested all 5 folds.

### Individual Explainer for Outcome Assessment

Feature importance lists the features that the model considers prominent, and their contribution scores, in the process of training the data using the tree-based algorithm model. However, we considered XGB as the final model not only because of its high performance but also because of the access to the decision-making process inside the model. By approaching the trees, it is possible to describe the specific features and their influences that contribute to the prediction of each patient’s daily prediction of discharge.

We demonstrate an individual explainer that can help in the interpretation of the XGB prediction results using a waterfall chart. Also called a bridge or cascade chart, it is a type of bar chart that portrays relative values and calculates the difference between adjacent values. It can show the positive or negative influence and gradual direction of the final discharge score.

To estimate values for individual explainers, we predicted the desired records with the trained XGB and obtained the contributions of all the features. The contribution refers to a feature’s influence obtained by aggregating the scores that each feature contributes to all trees. Subsequently, we calculated the logistic value—*logistics* (*x*) = 1 / (1 + *e^-x^*)—of the feature’s influence and the relative values required for the explainer. We selected the number of features to be displayed as 15, and the remaining 871 features were integrated and displayed simultaneously as *others* in the explainer.

## Results

### Data Characteristics

We created a data set that consisted of 669,667 records with 886 features, including diagnosis code, laboratory test results, physical information, medication, procedure, operation, PACS, and transfusion. Patients were admitted to cardiology or thoracic surgery, and their LOS ranged from 3 to 30 days. The average age of the patients was 61.03 (SD 13.42) years. The data set comprised 37.97% (254,254/669,667) women and 62.03% (415,413/669,667) men.

### Performance of the ML-Based Predictive Models

#### Final ML-Based Model Selection

**Figure 6:**
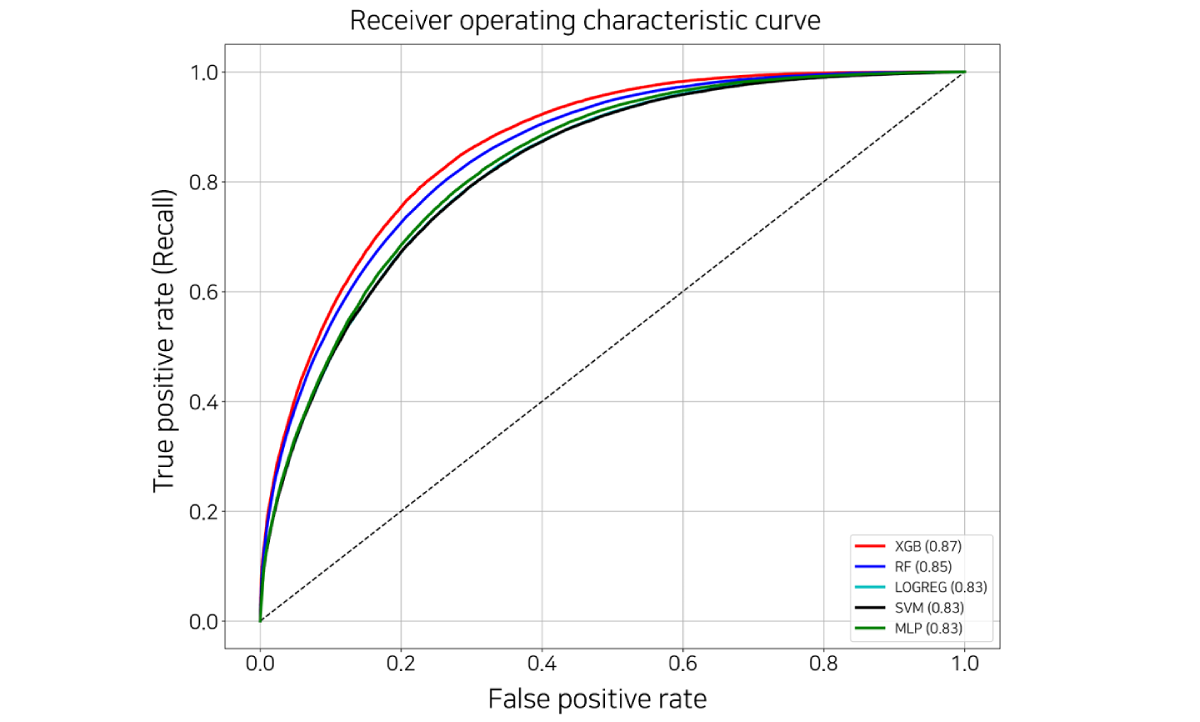
Receiver operating characteristic curve of the machine learning–based models. LOGREG: logistic regression; MLP: multilayer perceptron; RF: random forest; SVM: support vector machine; XGB: extreme gradient boosting.

We experimented with the 5 ML-based models using 5 cross-validations. The AUROC score for each fold is listed in Table 1. The highest AUROC score for each fold is shown in italics, and the *support* column in Table 1 represents the number of each true label. Figure 6 shows the ROC curve plot; the area of the curve is represented by the AUROC and has a value between 0 and 1. The closer the AUROC score is to 1, the higher the model performance. XGB achieved the highest and a relatively stable score on all folds. Table 2 provides a comparison of the 5 ML-based models. All scores in Table 2 are the average values of the results and the SD in 5 folds, and the highest score for each metric is shown in italics. The specificity of logistic regression and support vector machine, which obtained 0.828, was the highest, but XGB achieved the highest in the rest of the metrics. Particularly, although the label of the data set was imbalanced, XGB scored 0.7 or higher for predicting label 1. Hence, we chose XGB as the final model to predict discharge probability.

**Table 1:** Evaluation by area under the receiver operating characteristic score of 5-fold cross-validation for each model.

	LR^a^	SVM^b^	RF^c^	MLP^d^	XGB^e^	Support (0, 1)
Fold 1	0.826	0.825	0.853	0.833	*0.866* ^f^	(83,113, 50,188)
Fold 2	0.827	0.826	0.851	0.835	*0.868*	(83,538, 50,310)
Fold 3	0.824	0.824	0.850	0.821	*0.865*	(84,192, 50,585)
Fold 4	0.824	0.823	0.850	0.831	*0.864*	(83,969, 50,460)
Fold 5	0.822	0.821	0.848	0.834	*0.863*	(82,918, 50,394)
Value, mean (SD)	0.824 (0.002)	0.824 (0.002)	0.850 (0.002)	0.831 (0.005)	*0.865* (0.002)	N/A^g^

^a^LR: logistic regression.

^b^SVM: support vector machine.

^c^RF: random forest.

^d^MLP: multilayer perceptron.

^e^XGB: extreme gradient boosting.

^f^The italicized values indicate the highest score of each fold.

^g^N/A: not applicable.

**Table 2:** Comparison of the 5 machine learning–based models by metric.

Model	Values, mean (SD)					
	ACC^a^	Sen^b^	Spe^c^	PPV^d^	NPV^e^	AUROC^f^
LR^g^	0.75 (0)	0.624 (0.005)	*0.828*^h^ (0.004)	0.686 (0.005)	0.786 (0.005)	0.824 (0.002)
SVM^i^	0.75 (0)	0.624 (0.005)	*0.828* (0.004)	0.686 (0.005)	0.784 (0.005)	0.824 (0.002)
RF^j^	0.77 (0)	0.696 (0.005)	0.818 (0.004)	0.696 (0.005)	0.818 (0.004)	0.85 (0.002)
MLP^k^	0.758 (0.004)	0.642 (0.017)	0.822 (0.007)	0.686 (0.005)	0.792 (0.007)	0.831 (0.005)
XGB^l^	*0.782* (0.004)	*0.716* (0.005)	0.824 (0.005)	*0.71* (0)	*0.828* (0.004)	*0.865* (0.002)

^a^ACC: accuracy.

^b^Sen: sensitivity.

^c^Spe: specificity.

^d^PPV: positive predictive value.

^e^NPV: negative predictive value.

^f^AUROC: area under the receiver operating characteristic.

^g^LR: logistic regression.

^h^The italicized values refer to the highest score of each metric.

^i^SVM: support vector machine.

^j^RF: random forest.

^k^MLP: multilayer perceptron.

^l^XGB: extreme gradient boosting_._

**Figure 7:**
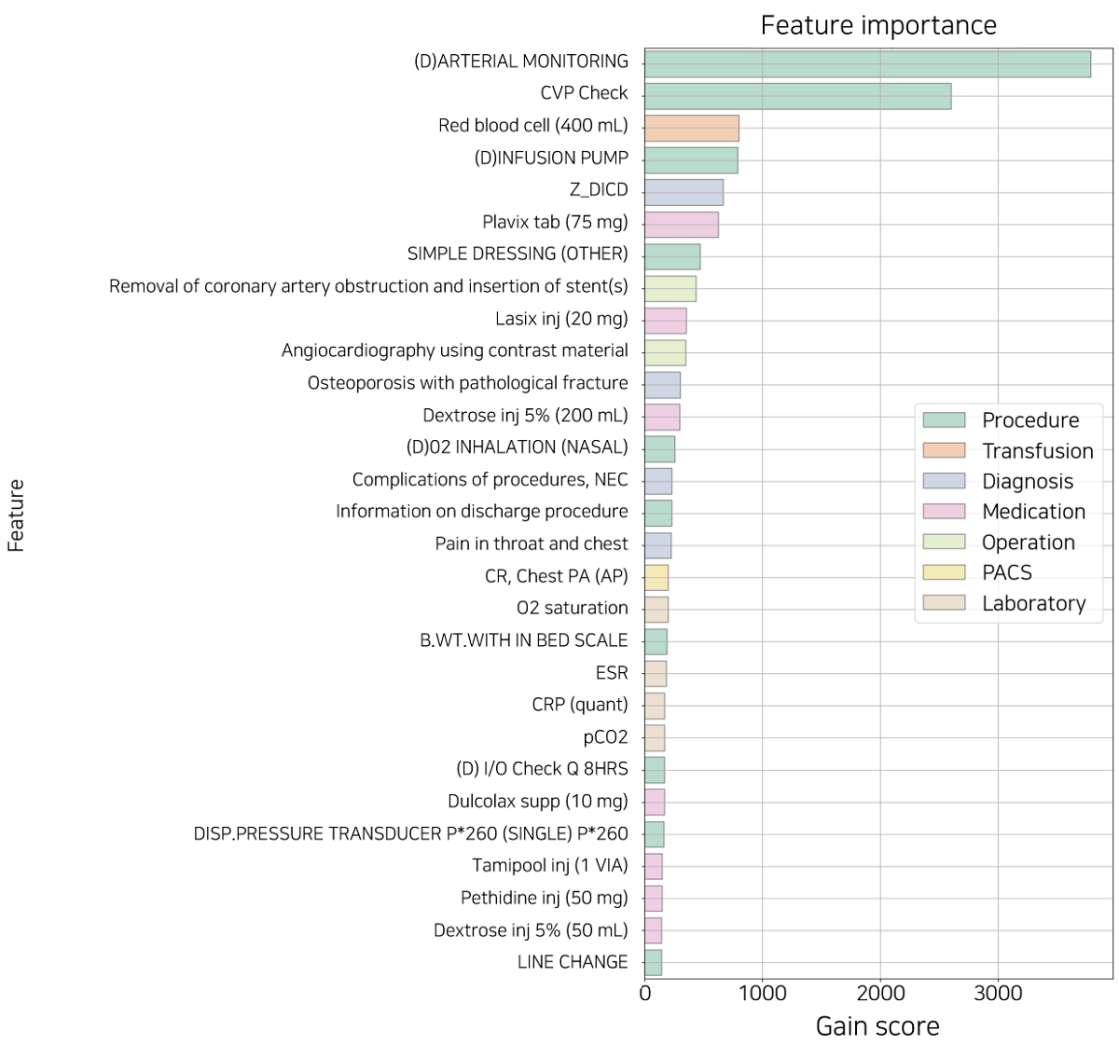
The feature importance sorted by gain score. B.WT.: body weight; CR: chest radiograph; CRP: C-reactive protein; CVP: central venous pressure; DISP: disposable; ESR: erythrocyte sedimentation rate; I/O: intake and output; supp: suppository; inj: injection; NEC: necrotizing enterocolitis; PA: posteroanterior; PACS: picture archiving and communication system; Z_DICD: all diagnostic codes not selected for one-hot encoding.

Figure 7 shows the relative feature importance of XGB sorted by gain score. The gain score refers to the average gain across all splits that the feature is used in. All the features used in the model have been replaced by their names used in the AMC. Except for the date-related feature, all other features that affected the model were found in all the tables. The features in the procedure table are substantially related to clinically critical situations. For example, the terms denoted with *(D)* are likely to mean a more severe state than others. The remaining features are also associated with CVDs or include primary examination and prescriptions during hospitalization.

However, because feature importance can only explain the model but not each patient, it is insufficient for use as an individual explainer for prediction. Depending on the patient’s condition, different features affect the daily probability of discharge. Therefore, we suggested an individual explainer that provides a patient-specific feature for daily prediction during hospitalization.

#### Feature Reduction

Too many features tend to reflect negatively on the model performance; therefore, it was necessary to select an appropriate number of features. We performed recursive feature elimination with cross-validation (RFECV). This algorithm aims to identify the optimal number of features by comparing model performance while eliminating the features with low feature importance one at a time. RFECV returns the ranks and names of all features; we identified approximately 150 features with a rank of 1 by applying RFECV to our final model XGB. For performance comparison, we performed 5-fold cross-validation using the same data set with the same parameters. The number of features to be compared was 886 (all), 150 selected by RFECV, and the top 50 features in the model trained with the 150 selected by RFECV.

**Figure 8:**
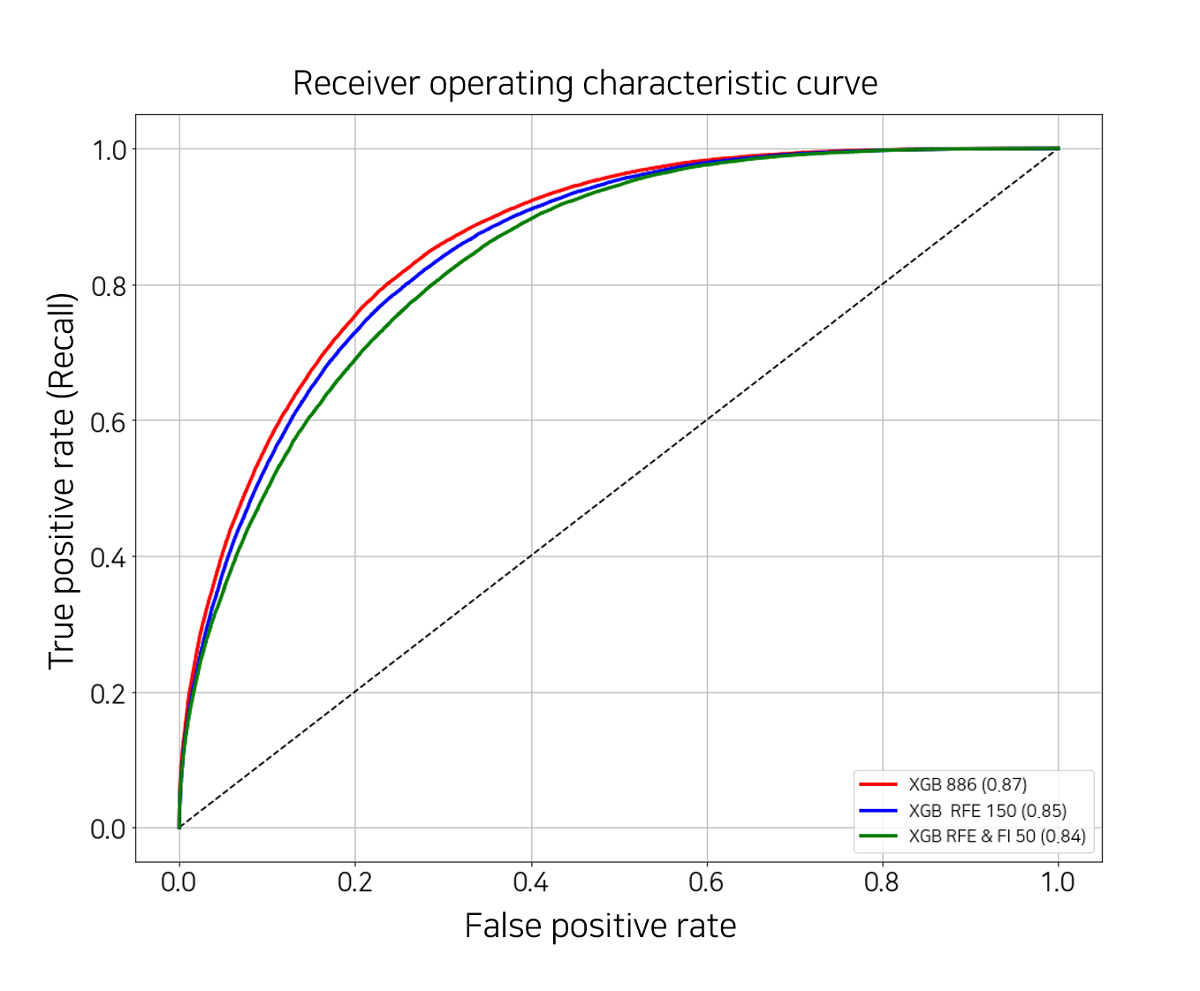
Receiver operating characteristic curve of the extreme gradient boosting models with the different number of features. FI: feature importance; RFE: recursive feature elimination; XGB: extreme gradient boosting.

As shown in Figure 8 and Table 3, the performance difference between the model using all the features and the models with 150 and 50 features was only approximately 1% to 2.5% based on the AUROC score. This indicates that even with 83.1% to 94.4% of feature reduction, there is only a maximum performance difference of 2.5%. Therefore, a suitable number of features should be selected considering the situation in each hospital or the data characteristic.

**Table 3:** Evaluation by area under the receiver operating characteristic (AUROC) score of 5-fold cross-validation to select features.

Number of features	Values, mean (SD)					
	ACC^a^	Sen^b^	Spe^c^	PPV^d^	NPV^e^	AUROC
886 (All)	*0.782*^f^ (0.004)	*0.716* (0.005)	*0.824* (0.005)	*0.71* (0)	*0.828* (0.004)	*0.865* (0.0018)
150 (RFE^g^)	0.77 (0)	0.696 (0.005)	0.814 (0.005)	0.694 (0.005)	0.818 (0.004)	0.853 (0.0018)
50 (RFE and FI^h^)	0.76 (0)	0.67 (0.006)	0.812 (0.004)	0.682 (0.004)	0.802 (0.004)	0.840 (0.00096)

^a^ACC: accuracy.

^b^Sen: sensitivity.

^c^Spe: specificity.

^d^PPV: positive predictive value.

^e^NPV: negative predictive value.

^f^The italicized values refer to the highest score of each metric.

^g^RFE: recursive feature elimination.

^h^FI: feature importance.

### Explainer of Individual Prediction for Outcome Assessment

#### Overview

The predictive model classifies the data as *0* or *1* based on a threshold. The optimal threshold is the point where the sum of sensitivity and precision can be maximized simultaneously (in the ROC curve, true-positive rate and false-positive rate are proportional to each other). However, sensitivity and precision require trade-off against each other; therefore, decreasing FN increases sensitivity, and decreasing false positive increases precision. In other words, it is necessary to adjust for the appropriate threshold to suit the decision point of the hospital operation.

We presented the daily discharge score during hospitalization and the influence of the features by date through the explainer of individualized predictions. The following section includes a description and an example of our explainer for the sample data set, which represents one of the patients in the test set.

#### Discharge Score During Hospitalization

**Figure 9:**
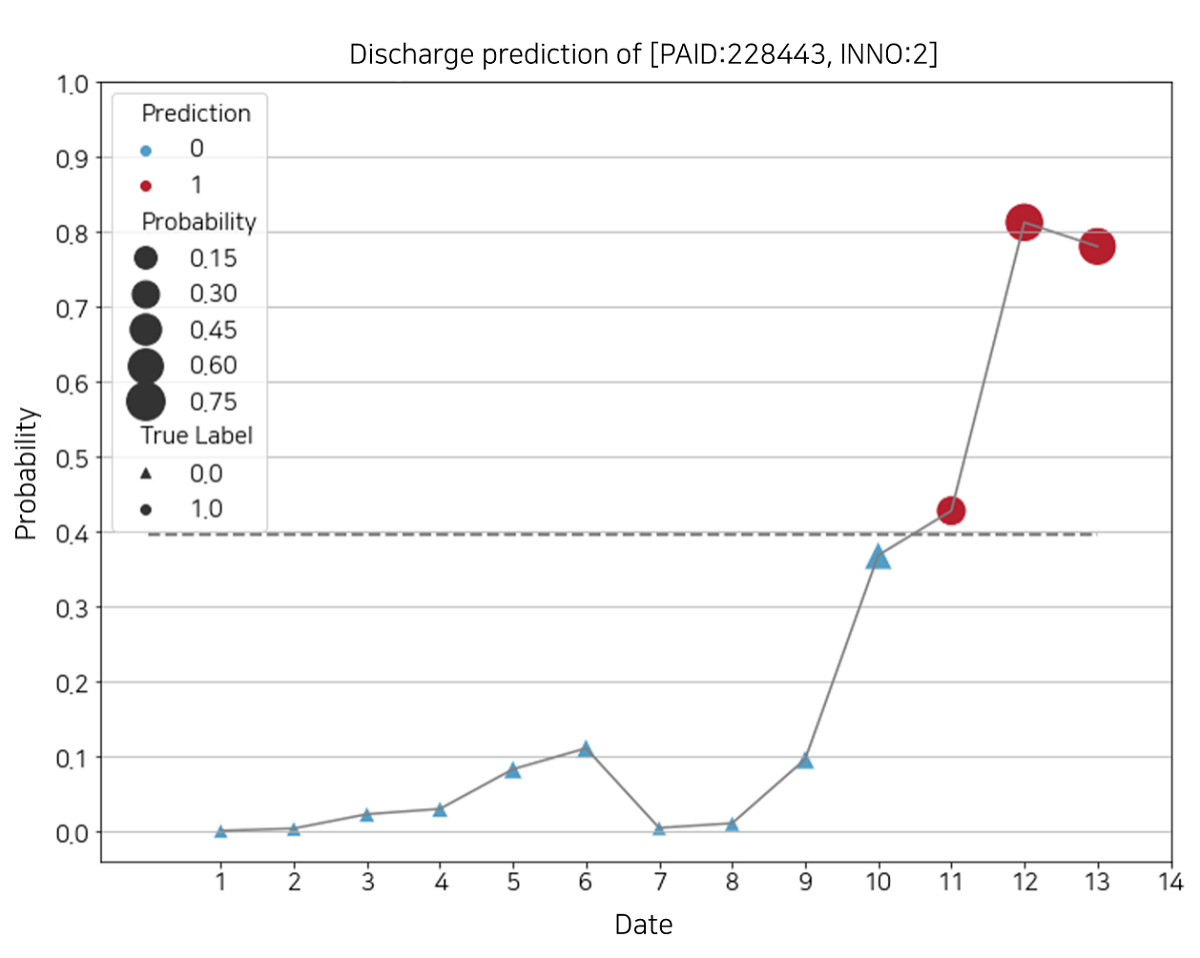
Daily discharge score of a patient’s identification of 228,443 and patient’s encounter number of 2. INNO: patient’s encounter number; PAID: patient’s identification.

The sample data set consisted of the records of a patient with a PAID of 228,443 and an INNO of 2, hospitalized for 13 days and discharged on day 14. The patient’s daily discharge score plot is depicted in Figure 9. The plot's x-axis represents the daily date excepted discharge date (ie, day 14) within the patient’s hospitalization period, and the y-axis represents the probability of discharge (ie, discharge score). The model’s optimal threshold was 0.39, indicated by a horizontal dotted line. The circle and the triangle represent the true labels *1* and *0*, respectively, and the size of the figure is proportional to the discharge score. The colors of the figure denote the results predicted by the model: red for positive prediction (label *1*, discharge) and blue for negative prediction (label *0*, admission).

For this sample, the model accurately predicted the discharge within 3 days. However, if the threshold is adjusted, the prediction results may change on dates 11 and 12. For example, if the current threshold rises slightly, *1* is applicable only for dates 12 and 13. This can be useful when trying to avoid false positive even if the false negative increases.

#### Daily Feature Influence Score

**Figure 10:**
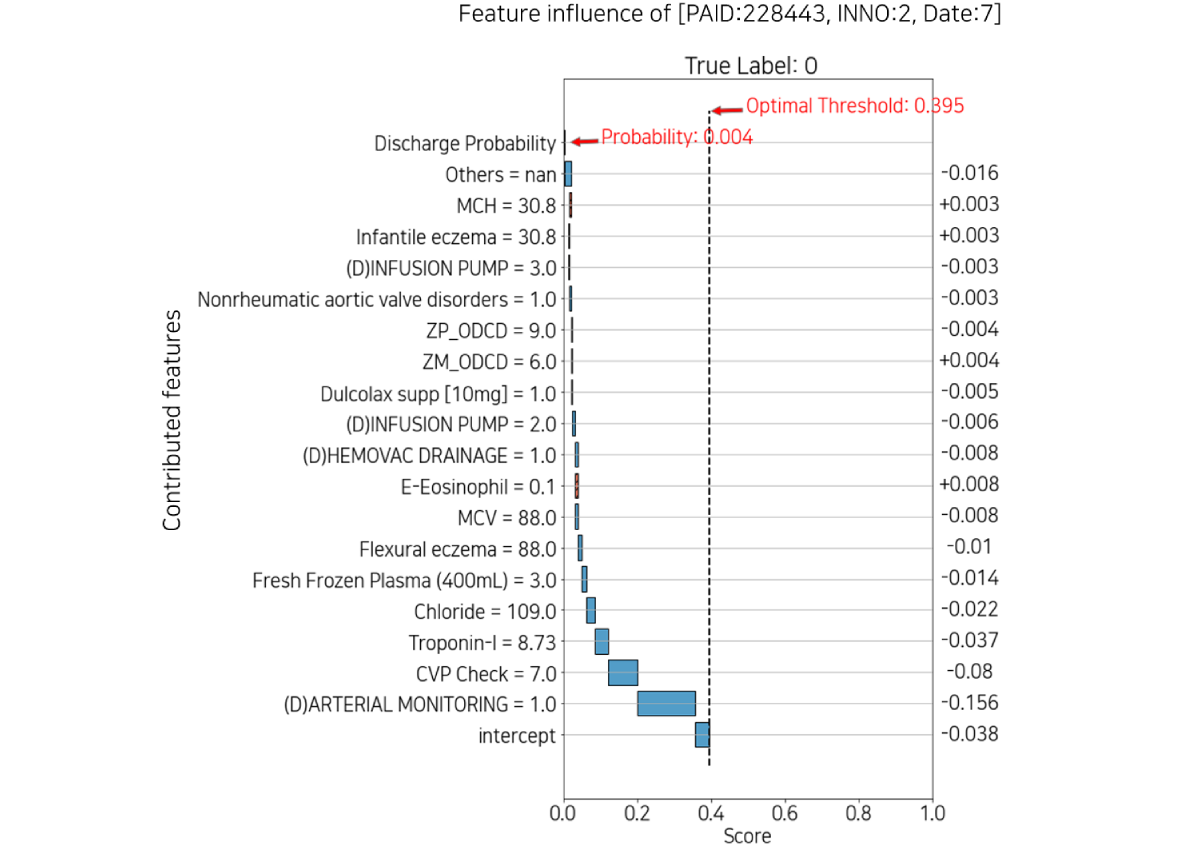
Feature influence with low probability of discharge date 7. CVP: central venous pressure; INNO: patient’s encounter number; MCH: mean corpuscular hemoglobin; MCV: mean corpuscular volume; PAID: patient’s identification; supp: suppository; ZM_ODCD: all medication codes not selected for one-hot encoding; ZP_ODCD: all procedure codes not selected for one-hot encoding.

**Figure 11:**
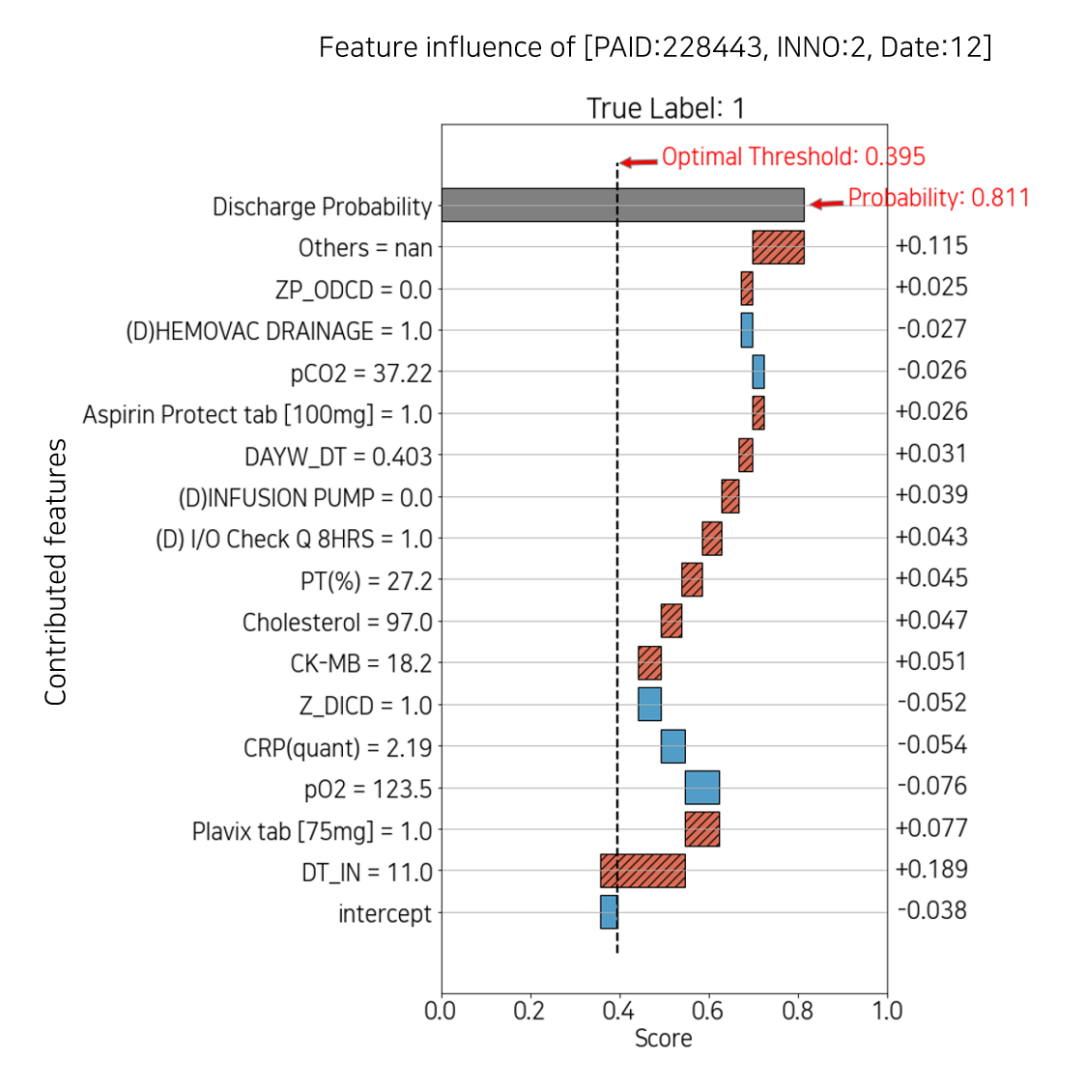
Feature influence with high probability of discharge on date 12. CK-MB: creatine kinase-myoglobin binding; CRP: C-reactive protein; DAYW_DT: integer feature of weekday; DT_IN: time since admission date in days; I/O: intake and output; INNO: the patient’s encounter number; PAID: the patient’s identification; PT: prothrombin time; Z_DICD: all diagnostic codes not selected for one-hot encoding; ZP_ODCD: all procedure codes not selected for one-hot encoding.

Figures 10 and 11 describe the plot of feature influence for each day. The following is the basic description of the individual explainer: the x-axis of the plot is a score ranging from 0 to 1, and the y-axis represents the contributed features and the corresponding values that influenced the probability of discharge on that day. The threshold represented by the vertical dotted line is equal to the optimal threshold in Figure 9. The intercept, the plain blue box at the bottom of the y-axis, is a revised value reflecting that the number of each true label is imbalanced. The discharge probability, the gray box at the top of the y-axis, is the discharge score, which is the same as the probability in Figure 9. The width of each box corresponding to the feature refers to the absolute value of each score. The original score is indicated on the right side of the plot. The absolute value decreases from bottom to top, which means the contribution to the discharge score also decreases (the box of *others* is relatively wide because it is the sum of the scores of approximately 800 features, excluding the features below it). The red box with diagonal hatching represents each score of the feature that positively contributed to the discharge score and moves to the right. Conversely, the plain blue box represents negatively contributing feature scores and moves to the left.

To summarize, on the y-axis, from bottom to top, the features contributed to the prediction; the diagonal hatched red box to the right is positive, and the plain blue box to the left is negative.

Figure 10 shows the feature influence at day 7 with a low probability of discharge of 0.004, and Figure 11 shows day 12 with a high probability of 0.811. In Figure 10, *arterial monitoring=1* and *infusion pump=3* negatively affected the probability. In contrast, in Figure 11, *infusion pump=0* had a positive effect on probability. Because arterial monitoring and infusion pump are mainly prescribed for critical patients, both consist mostly of zeros in the data set. Therefore, displaying features and values together can help the medical staff interpret the plot intuitively. Moreover, each explainer may or may not have the features that appeared in the feature importance plot. This suggests that it is also necessary to identify features that contributed to individual patients rather than managing only the features of feature importance.

### Outcome Assessment

Figure 1 shows the simulated impact in bed management applied with our predictive model and individual explainer. It is possible to recognize the probabilities of discharge of all patients for each ward every day. The paramount features and values that affect the discharge scores can be identified at once. It is informative for interpreting both high or low probability because the explainer implies the reasoning not only for discharge but also prolonged discharge. Similarly, it is possible to obtain information based on the expected discharge date of each patient, such as bed capacity in the near future. For the human and physical resources of the hospital to be used efficiently, future bed availability information can help reduce hospital costs through better management of beds and hospitalization reservations.

## Discussion

### Principal Findings

Investigations into bed management, which requires the use of hospital processes, and biomarker detection for patient-specific care, are actively pursued. In this study, we propose an ML-based predictive model to identify the discharge date for better bed management and the risk factors regarding discharge and CVDs. However, because each hospital has varying environmental variables, an algorithm that can consider them collectively was needed. Our study can contribute to improving the algorithm and supporting health care services. We have summarized the expectations of our predictive model and its explanation, along with its limitations.

First, we predicted the possibility of discharge to learn future information, but for the model to be practically applied, objective information about the current bed situation must be obtained. Currently, we are collecting bed information to combine it with the prediction results and optimize overall bed management. Consequently, our predictive model can be extended from ward-level up to hospital-level bed management. It may reduce the labor-intensive tasks for the medical team and the waiting time for patients.

Second, although our model provides adjustment of the optimal threshold according to the hospital circumstances, the ambiguity of decision-making because of results near the threshold exists, such as dates 10 and 11 in Figure 9. To solve this problem, there is a method that uses weighted average to make the result more conservative but reliable. Instead of using the probability returned by the model directly, it may be more useful to use it after weighting it for the past results, so that the target day reflects the weighted past results. It is just as necessary to produce reliable results as it is trying to explain the model and its internal features.

Finally, EHRs are longitudinal and sequential, but the sequence is different for each patient, and they do not have a regular interval. Consequently, we are preparing a preprocessing technique that can properly control the EHRs and reflect them in the model. Furthermore, compared with computer visualization, sequential data are relatively difficult to apply to XAI. Still, we are preparing explainable methods that are compatible with these data.

### Conclusions

In this study, we have proposed an ML-based model to predict the daily discharge probability for each patient and demonstrated the individual explainer for any date during hospitalization, along with the reasonable contributing features. Our XGB model accomplished an AUROC of 0.865 and represented the simulated bed management based on explainable features. It could assist the medical team and patients in identifying the individual and common risk factors in CVDs and support hospital administrators in improving the management of hospital beds and other resources.

## Abbreviations

AMC: Asan Medical Center
AUROC: area under the receiver operating characteristic
CVD: cardiovascular disease
EHR: electronic health record
GBM: gradient boosting algorithm
ICU: intensive care unit
INDT: date of visitation or admission
INNO: patient encounter number
LOS: length of stay
ML: machine learning
OHE: one-hot encoding
OUDT: date of discharge
PAID: patient identification
RFECV: recursive feature elimination with cross-validation
ROC: receiver operating characteristic
XAI: explainable artificial intelligence
XGB: extreme gradient boosting
